# Epidemiological characteristics of neuroendocrine neoplasms in Beijing: a population-based retrospective study

**DOI:** 10.1186/s12889-024-18845-8

**Published:** 2024-05-24

**Authors:** Yujia Chi, Shuo Liu, Jianwei Zhang, Huichao Li, Lei Yang, Xi Zhang, Haoxin Li, Qingyu Li, Ning Wang, Ming Lu, Minglei Zhuo

**Affiliations:** 1 Key Laboratory of Carcinogenesis and Translational Research (Ministry of Education/Beijing), Department I of Thoracic Oncology, Peking University Cancer Hospital & Institute, 52 Fucheng Road, Haidian District, Beijing, 100142 China; 2 Key laboratory of Carcinogenesis and Translational Research (Ministry of Education/Beijing), Beijing Office for Cancer Prevention and Control, Peking University Cancer Hospital & Institute, Beijing, 100142 China; 3 Key Laboratory of Carcinogenesis and Translational Research (Ministry of Education/Beijing), Department of Gastrointestinal Oncology, Peking University Cancer Hospital & Institute, 52 Fucheng Road, Haidian District, Beijing, 100142 China

**Keywords:** Neuroendocrine neoplasms (NENs), Epidemiological characteristics, Incidence, Overall survival

## Abstract

**Background:**

The incidence of neuroendocrine neoplasms (NENs) is rising rapidly worldwide. However, there are few reports on these heterogeneous diseases in China. Our study aimed to explore the epidemiological characteristics of NENs in Beijing.

**Methods:**

We conducted a retrospective cohort study using population-based cancer surveillance data in Beijing, China. All data were extracted from the Beijing Cancer Registry with incidence dates from 1 January 1998 to 31 December 2018; the follow-up period was through 31 December 2021. Segi’s world standard population was used to estimate the age-standardized rate. Survival was estimated using the Kaplan–Meier method.

**Results:**

From 1998 to 2018, the incidence of NENs in Beijing initially showed a significant increasing trend, from 1.07/100,000 to 3.53/100,000; this began to plateau after 2013. The age-specific incidence rate increased with age and peaked in the age group 70–74 years. The incidence in men was significantly higher than that in women (4.41/100,000 vs. 1.69/100,000). The most common sites of NENs were the lung (2.38/100,000) and rectum (0.14/100,000). Most NENs were diagnosed at a late stage. We found that NENs originating from the lung had worse overall survival than extrapulmonary NENs, and male patients had worse survival than female patients.

**Conclusions:**

This study retrospectively analyzed the epidemiological characteristics of NENs in Beijing from 1998 to 2018. Our findings provide a reference regarding the epidemiological statistics of NENs in Beijing to contribute to the prevention, diagnosis, and treatment of these specific tumors.

**Supplementary Information:**

The online version contains supplementary material available at 10.1186/s12889-024-18845-8.

## Background

Neuroendocrine neoplasms (NENs) constitute a heterogeneous and rare group of tumors arising from neuroendocrine cells, accounting for a very small portion of gastrointestinal and bronchial malignancies [1–4]. The most common origin sites include the gastrointestinal tract, lungs, and pancreas. Though relatively rare, NENs can also originate in the ovary, thyroid gland, parathyroid gland, pituitary gland, and adrenal gland. NENs can exhibit a variety of hormonal syndromes owing to hormone production, such as carcinoid syndrome, Verner–Morrison syndrome caused by vasoactive intestinal peptide secreting tumor, 4D syndrome caused by glucagonoma, Zollinger-Ellison syndrome caused by gastrinoma, insulinoma, Cushing’s syndrome, and acromegaly [5]. It is reported that most NENs are neuroendocrine tumors (NETs) and some can possess an indolent disease biology. However, G2/3 NETs and neuroendocrine carcinomas (NECs) have also been reported, which are highly proliferative tumors characterized by rapid disease progression [6].

NENs were discovered in the early 20th century. As the understanding of NENs has deepened over the past 100 years, the disease classification has been updated several times. Owing to the rarity, complexity, and heterogeneity of these diseases, our understanding of NENs is still very limited. NENs remain difficult to diagnose, and misdiagnosis and delayed diagnosis are common problems, resulting in missing the opportunity to provide the most appropriate treatment. Because of hormone secretion during disease progression, various symptoms may affect organ function and even cause sequelae, seriously affecting patients’ quality of life and consuming valuable medical resources. Globally, medical experts have intensified their research on NENs in recent years. The means of examination are becoming more targeted, and various new drugs, including somatostatin analogues and targeted biological therapy, have been discovered, with substantial survival benefits to patients [7]. However, it is important to note that despite these advancements, the incidence of NENs is generally on the rise around the world, although there are significant differences among different regions. In European and American countries, the incidence of NENs is relatively high whereas it is relatively low in Asian countries. The standardized incidence rate of NETs was 0.24/100,000 in 1996 and rose to 3.16/100,000 in 2015 in Taiwan, China [8]; this rate for gastroenteropancreatic NENs (GEP-NENs) in Japan was 3.53/100,000 in 2016 [9]. However, NENs currently lack comprehensive statistics and large-scale reports, highlighting the need for more in-depth investigation into these tumors.

In this study, we examined the epidemiological characteristics of NENs using population-based cancer registry data and calculated the age-standardized incidence rates stratified by residential area, cancer type, and site based on the characteristics of NENs. Our study aimed to fill the gap in this field by analyzing the incidence and survival of NENs in different populations and time periods. This study can also provide a comprehensive epidemiological profile of NENs in China and contribute to the development of prevention and treatment strategies for these diseases.

## Materials and methods

### Data source

Beijing is the capital of China and is located in the North China region. The registered household population of Beijing is approximately 14 million, with nearly 20% of the population aged over 65 years. There are 16 districts in Beijing, of which six are classified as urban areas, accounting for 60% of the total population; the remaining 10 districts are classified as rural areas. All data analyzed in this study were extracted from the Beijing Cancer Registry (BCR). The BCR is a population-based cancer registry that began collecting cancer surveillance data in 1977 and has covered the total population of Beijing since 1998. The data quality is high, with BCR data included in the Cancer Incidence in Five Continents report by the International Agency for Research on Cancer (IARC). The variables in the BCR include personal information, diagnostic information, and follow-up information. Stage data were collected from 2012 in the BCR. The rules published by the International Agency for Research on Cancer/International Association of Cancer Registries (IARC/IACR) were followed to define the date of the first consultation at, or admission to, a hospital, clinic, or institution for cancer as to the incidence date. The date of the first cancer diagnosis made by a physician or date of the first pathology report was used if the admission date was unavailable [10].

In this study, the incidence data from 1 January 1998 to 31 December 2018 were included. The range of codes extracted from the database was in accordance with the International Classification of Diseases for Oncology 3rd Edition (ICD-O-3.2) morphology codes. The ICD-O-3.2 codes in this study were 8002, 8013, 8040–8045, 8150–8156, 8240–8246, 8249. Paraganglioma (8680,8693), pheochromocytoma (8700), and medullary carcinoma (8510) of the thyroid were excluded from this study.

The population data stratified by age, sex, and residential area were provided by the Beijing Municipal Public Security Bureau. This study covered 256,811,955 person-years (129,250,688 for male individuals and 127,561,267 for female individuals, 157,699,572 for urban areas, and 99,112,383 for rural areas). We used both passive and active follow-up methods to obtain the vital status of patients with cancer. The mortality database of patients with cancer, provided by the Beijing Center for Disease Prevention and Control (Beijing CDC), was used to link to the cancer registry incidence database by identifiable information. Unlinked patients were further followed by community health cancer/station staff through telephone calls or family visits. The last day of follow-up was 31 December 2021.

### Quality control

Medical record re-abstraction and annual training for cancer registrars in hospitals were implemented regularly to ensure the standardization of data coding and improve data quality. IARC/IACR Cancer Registry Tools was used to check whether the variables were complete, accurate, and logical [11]. For cases marked with a warning or error by the software, we contacted the original reporting hospital to check the medical records directly. All NEN cases included in this study were morphologically verified; none was a death certificate-only case. The rate of patient loss to follow up was 2.60%.

### Statistical analysis

Pearson’s chi-squared test and the Kruskal–Wallis test were applied to calculate whether the differences in general information (sex, residential area, treatment hospital, age of onset, and race ethnicity) by cancer type statistics were significantly based on the data distribution. Neuroendocrine tumor incidence rates were stratified by age (0–84 by 5 year intervals and 85 years and older), sex (male and female), residential area (urban and rural), cancer site and histology type (small cell lung carcinoma [SCLC] and pulmonary large cell neuroendocrine carcinoma [LCNEC], pulmonary carcinoid tumor, extrapulmonary NEC, and extrapulmonary NET), site (lung, stomach, small intestine, colon, rectum, pancreas, and esophagus), and embryonic digestive tube (foregut, midgut, and hindgut). The age-standardized incidence rates were calculated using Segi’s world standard population as the standard world population structure. The stage standard was based on the TNM (tumor, node, metastasis) classification of malignant tumors, 7th Edition of the *AJCC Cancer Staging Manual* of the American Joint Committee on Cancer. Stage I and stage II were merged into early stage, and stage III and stage IV; were merged into advanced stage. Royston’s p trend and the Cochran–Armitage test were used to describe the change in proportion of early-stage cases. The observed survival (OS) was calculated using the Kaplan–Meier method. The OS was stratified by sex, residential area, cancer type, and stage. The log-rank test was used to assess whether the survival distribution was statistically significant. The average annual percentage change (AAPC) in the incidence rate was calculated using the Joinpoint Regression Program, version 4.9.0.0 (Statistical Research and Applications Branch, National Cancer Institute). All other statistical analyses were performed in Stata, version 15.1 (StataCorp LLC., College Station, TX, USA).

## Results

During the period 1998–2018, 12,896 new cases of NEN with identified histology subtypes were reported in Beijing, including 9602 cases of SCLC and pulmonary LCNEC, 738 cases of typical and atypical carcinoids originating from the lung or mediastinum, 1735 cases of extrapulmonary NEC, and 821 cases of extrapulmonary NET, respectively accounting for 74.46%, 5.72%, 13.45%, and 6.37% of the total. Median age at diagnosis of all NENs was 65 years. There were 9209 (71.41%) male patients and 3687 (28.59%) female patients, and the sex ratio was 2.5:1. The proportions by sex were statistically significant for different tumors. For example, the proportion of male patients with SCLC and LCNEC was significantly higher than that of female patients (75.63% vs. 24.37%). The proportions of male patients with extrapulmonary NEC and extrapulmonary NET were 57.93% (z = 15.02, *P* < 0.01) and 53.35% (z = 13.51, *P* < 0.01), respectively (Table 1); 8546 (66.27%) patients resided in urban areas and 4350 (33.73%) resided in rural areas; 12,241 patients (94.92%) were diagnosed in tertiary hospitals; and 95.64% of patients were Han ethnicity (Table 1).

**Table 1:** Distribution of NENs among patients in Beijing by primary tumor site and histology type, 1998–2018

	All NENs	SCLC&LCNEC	Pulmonary carcinoid tumor	Extrapulmonary NEC	Extrapulmonary NET	*P* value
Distribution, n(%)	12,896 (100.00)	9602 (74.46)	738 (5.72)	1735 (13.45)	821 (6.37)	
Age at NEC diagnosis,						
mean ± SD	63.42 ± 11.77	64.45 ± 10.76	62.14 ± 12.07	61.76 ± 14.18	56.06 ± 13.88	χ^2^ = 318.24, *P* < 0.01
median^a^	65	65	63	63	56	
Male sex^a^, n(%)	9209 (71.41)	7262 (75.63)	504(68.29)	1005(57.93)	438(53.35)	χ^2^ = 372.97, *P* < 0.01
Urban^a^, n(%)	8546(66.27)	6161(64.16)	520(70.46)	1228(70.78)	637(77.89)	χ^2^ = 87.68, *P* < 0.01
Tertiary^a^, n(%)	12,241 (94.92)	9080 (94.56)	710 (96.21)	1659 (95.62)	792 (96.47)	χ^2^ = 10.90, *P* = 0.01
Ethnic Han^a^, n(%)	12,334 (95.64)	9248 (96.31)	692 (93.77)	1621 (93.43)	773 (94.15)	χ^2^ = 41.35, *P* < 0.01

*NEN* Neuroendocrine neoplasm, *NEC* Neuroendocrine carcinoma, *NET* Neuroendocrine tumor, *SD* Standard deviation, *SCLC* Small cell lung cancer, *LCNEC* Large cell neuroendocrine carcinoma

^a^Statistically significant

During the period 1998–2018, in lung LCNEC and SCLC, the proportion of male patients was significantly higher than that in the other NENs (75.63% vs. 59.11%, *P* < 0.001); no significant correlation with sex was seen in the incidence rate of extrapulmonary NENs (χ2 = 4.75 *P* = 0.029; Notes: The significance level used for multiple comparisons was 0.008 after adjustment) (Table 1). There were significantly more newly diagnosed male patients than female patients in each year, and the annual incidence rate of male was more than twice that of female patients every year. Regarding the number of newly diagnosed cases, there were more cases in urban than in rural areas each year, but the gap in incidence rate decreased from 1998 to 2018 as the rural incidence rate increased rapidly (Table 2).

**Table 2:** Cases and rates of NENs stratified by sex and residential area from 1998 to 2018 in Beijing, China

Year	Male			Female			Urban			Rural			Total		
	cases	Incidence rate (1/100 000)	ASIRW (1/100 000)	cases	Incidence rate (1/100 000)	ASIRW (1/100 000)	cases	Incidence rate (1/100 000)	ASIRW (1/100 000)	cases	Incidence rate (1/100 000)	ASIRW (1/100 000)	cases	Incidence rate (1/100 000)	ASIRW (1/100 000)
1998	103	1.87	1.50	46	0.86	0.65	149	2.31	1.70	0	0.00	0.00	149	1.37	1.07
1999	129	2.33	1.81	49	0.91	0.69	174	2.67	1.89	4	0.09	0.08	178	1.63	1.24
2000	187	3.36	2.51	82	1.51	1.09	194	2.94	2.01	75	1.70	1.35	269	2.45	1.79
2001	202	3.60	2.63	93	1.70	1.19	228	3.41	2.28	67	1.52	1.17	295	2.66	1.90
2002	184	3.24	2.28	90	1.62	1.09	200	2.95	1.86	74	1.67	1.30	274	2.44	1.67
2003	283	4.94	3.45	126	2.24	1.51	327	4.76	3.01	82	1.83	1.40	409	3.61	2.46
2004	319	5.49	3.75	142	2.49	1.60	363	5.19	3.19	98	2.16	1.62	461	4.00	2.66
2005	363	6.13	4.03	127	2.19	1.39	352	4.92	2.90	138	3.01	2.18	490	4.18	2.68
2006	431	7.18	4.60	157	2.66	1.68	409	5.62	3.27	179	3.87	2.71	588	4.94	3.10
2007	469	7.72	4.92	167	2.80	1.68	439	5.94	3.38	197	4.23	2.95	636	5.28	3.25
2008	460	7.47	4.69	204	3.37	1.99	439	5.84	3.23	225	4.79	3.35	664	5.44	3.30
2009	499	8.00	4.77	231	3.76	2.17	465	6.08	3.30	265	5.60	3.64	730	5.90	3.44
2010	511	8.11	4.78	193	3.10	1.78	460	5.93	3.26	244	5.13	3.19	704	5.62	3.24
2011	566	8.88	5.19	239	3.79	2.11	517	6.56	3.54	288	6.01	3.74	805	6.35	3.61
2012	656	10.14	5.82	267	4.17	2.25	564	7.02	3.67	359	7.41	4.47	923	7.17	3.99
2013	646	9.85	5.77	288	4.42	2.36	588	7.20	3.89	346	7.06	4.23	934	7.15	4.03
2014	617	9.29	5.16	267	4.04	2.17	528	6.37	3.37	356	7.18	4.06	884	6.67	3.63
2015	603	8.99	4.90	205	3.07	1.53	501	5.98	3.02	307	6.12	3.43	808	6.03	3.17
2016	599	8.84	4.63	222	3.28	1.56	510	6.03	2.93	311	6.11	3.28	821	6.06	3.06
2017	655	9.64	4.85	226	3.31	1.68	543	6.43	3.05	338	6.54	3.46	881	6.47	3.21
2018	727	10.68	5.35	266	3.87	1.84	596	7.07	3.27	397	7.58	3.91	993	7.26	3.53
Total	9,209	7.12	4.41	3,687	2.89	1.69	8,546	5.42	3.07	4,350	4.39	2.98	12,896	5.02	3.01
AAPC %	9.11	7.82	5.45	8.19	6.74	4.30	6.61	5.01	2.87	22.09	14.48	11.54	8.85	7.47	5.08
95%CI	7.30-10.95	6.03–9.64	3.75–7.19	6.18–10.24	4.74–8.77	2.38–6.25	5.14–8.11	3.59–6.46	1.56–4.20	12.48–32.51	8.55–20.72	5.85–17.54	7.02–10.70	5.66–9.31	3.35–6.84
*P* value	< 0.001	< 0.001	< 0.001	< 0.001	< 0.001	< 0.001	< 0.001	< 0.001	< 0.001	< 0.001	< 0.001	< 0.001	< 0.001	< 0.001	< 0.001

*NEN* Neuroendocrine neoplasm, *ASIRW* Age standardized incidence rate by world standard population, *AAPC* Average annual percentage change

### Incidence

**Fig. 1:**
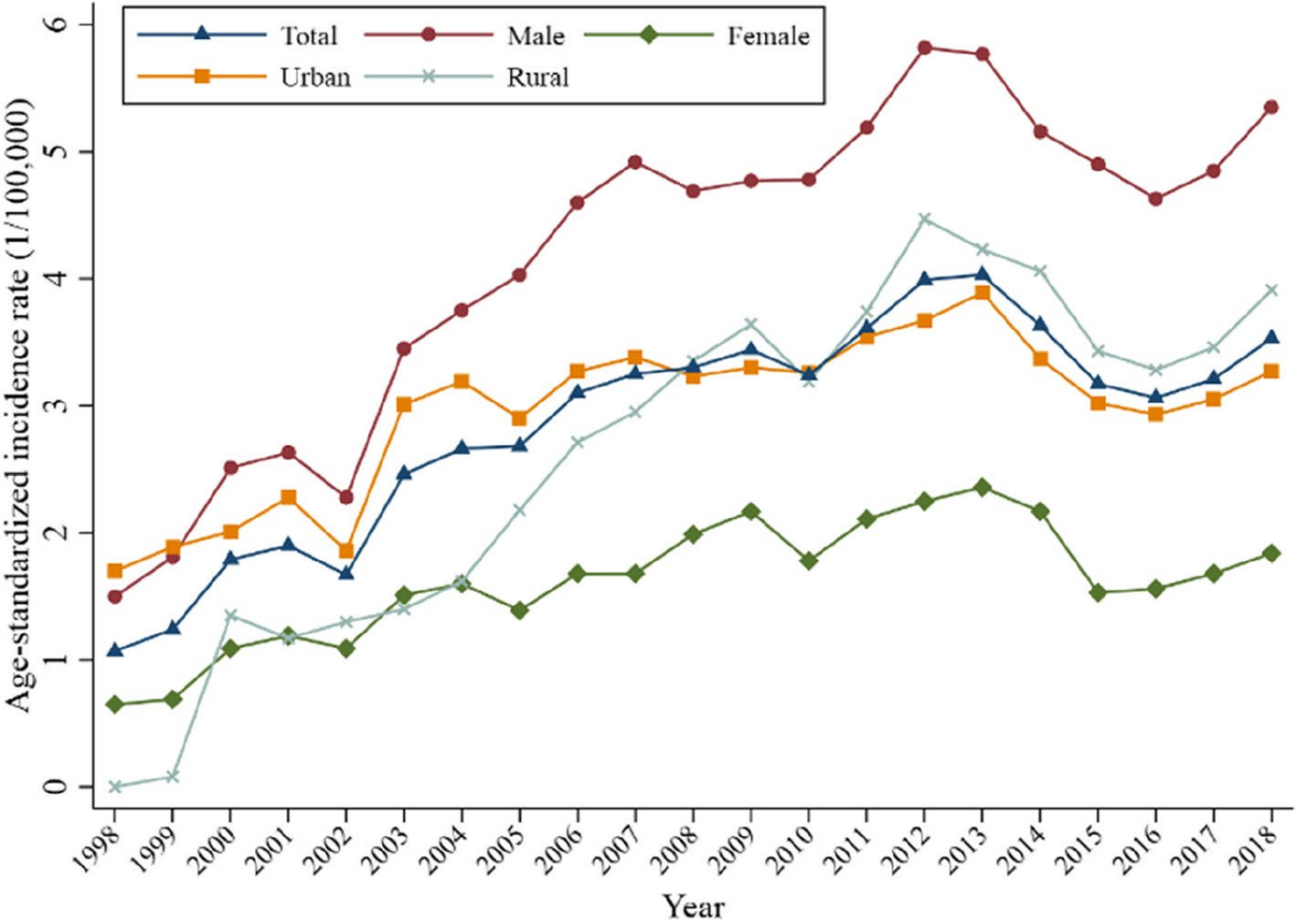
Age-standardized incidence rate of neuroendocrine neoplasms stratified by sex and residential area from 1998 to 2018 in Beijing, China

During the period 1998–2018, the number of newly diagnosed cases of NENs in Beijing showed an increasing trend each year, from 1.37 cases per 100,000 per year in 1998 to 7.26 cases per 100,000 per year in 2018 (Table 2). When standardized by age, the incidence rate of NENs in Beijing initially showed a significant increasing trend and began to plateau after 2013 (Fig. 1). The incidence increased from 1.07 cases per 100,000 per year in 1998 to 4.03 cases per 100,000 per year in 2013, which represented a 3.77-fold increase. In the following 5 years, the incidence fluctuated from 3.06/100,000 to 3.53/100,000. In terms of the overall population, according to age-standardized incidence rate by world standard population (ASIRW), a period of rapid growth occurred from 1998 to 2007, with an AAPC of 12.82% (95% confidence interval [CI]: 9.62–16.12%, *P* < 0.001), which essentially plateaued from 2007 to 2018, with an AAPC of − 0.38% (95% CI: −2.47–1.77%, *P* = 0.713).

The ARISW in men was significantly higher than that in women, with 4.41/100,000 versus 1.69/100,000 (Table 2). The AAPC of the ASIR for men (5.45%; 95% CI: 3.75–7.19%) was higher than that for women (4.30%; 95% CI: 2.38–6.25%), respectively. As seen in Fig. 1, the incidence in male patients was always higher than that in female patients, and the gap increased with time, with the male-to-female ASIRW ratio increasing from 2.31 in 1998 to 2.91 in 2018. The rising trend in rural incidence was obviously higher than that in urban incidence, and the gap narrowed gradually in the first decade; the suburban incidence exceeded that of urban incidence and maintained a certain gap in the second decade.

**Fig. 2:**
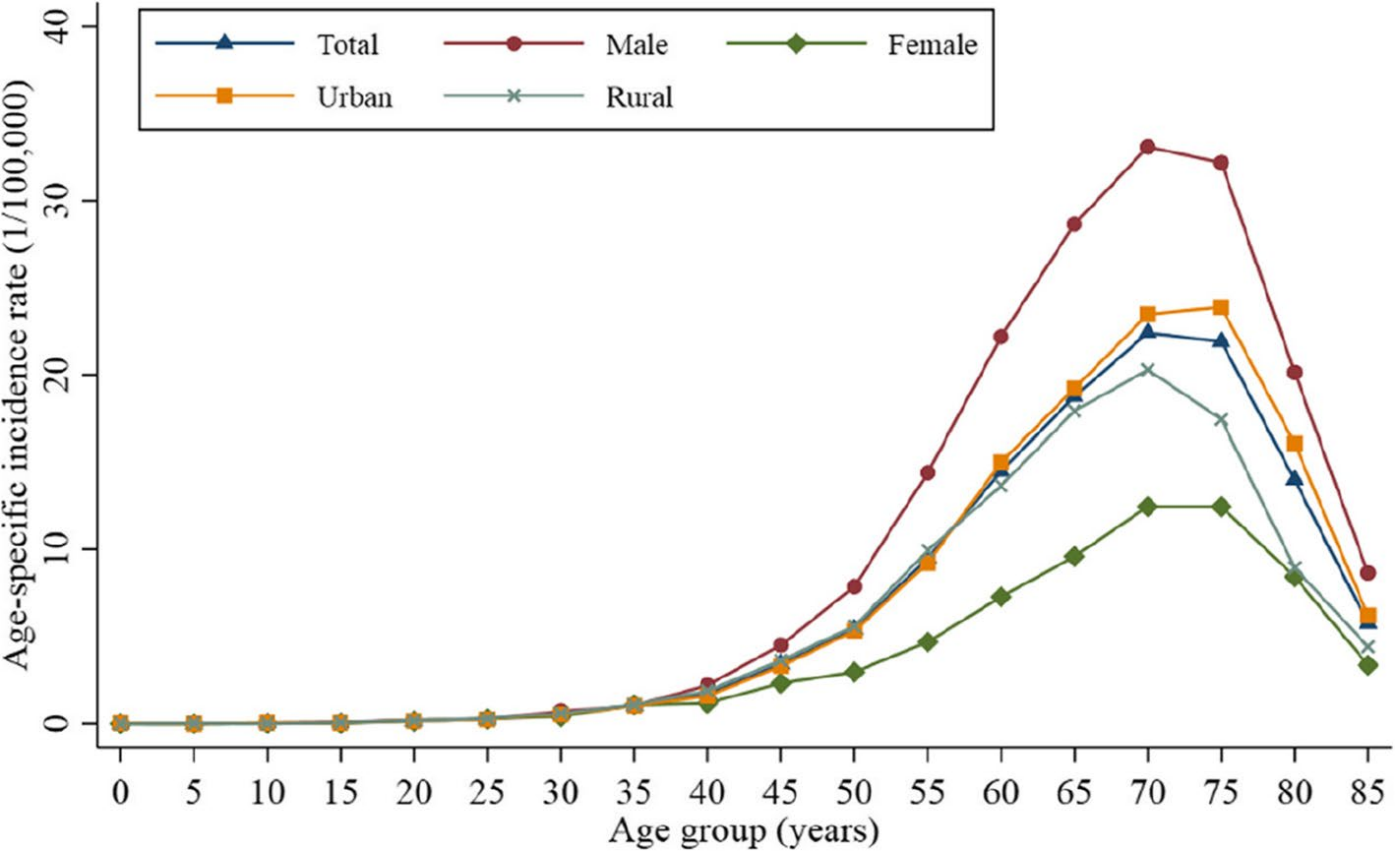
Age-specific incidence rate of neuroendocrine neoplasms stratified by sex and residential area from 1998 to 2018 in Beijing, China

The incidence of NENs was also significantly correlated with increasing age. The age-specific incidence rate increased with age and peaked in the age group 70–74 years. In the age group under 35 years, the incidence in each group was not more than 0.56/100,000; this gradually increased after age 45 years, reaching the highest level (22.41/100,000) in the age group 70–74 years and then gradually decreasing with increased age. This trend was not affected by the patient’s sex or the region where they were registered (Fig. 2). The average age among different pathological types and origin sites showed statistically significant differences. The median age of SCLC and LCNEC was 65 years, older than that of other NENs. The median age of pulmonary carcinoid tumor was similar to that of extrapulmonary NEC, which was 63 years old. The median age of extrapulmonary NET was younger, at age 56 years (Table 1).

**Fig. 3:**
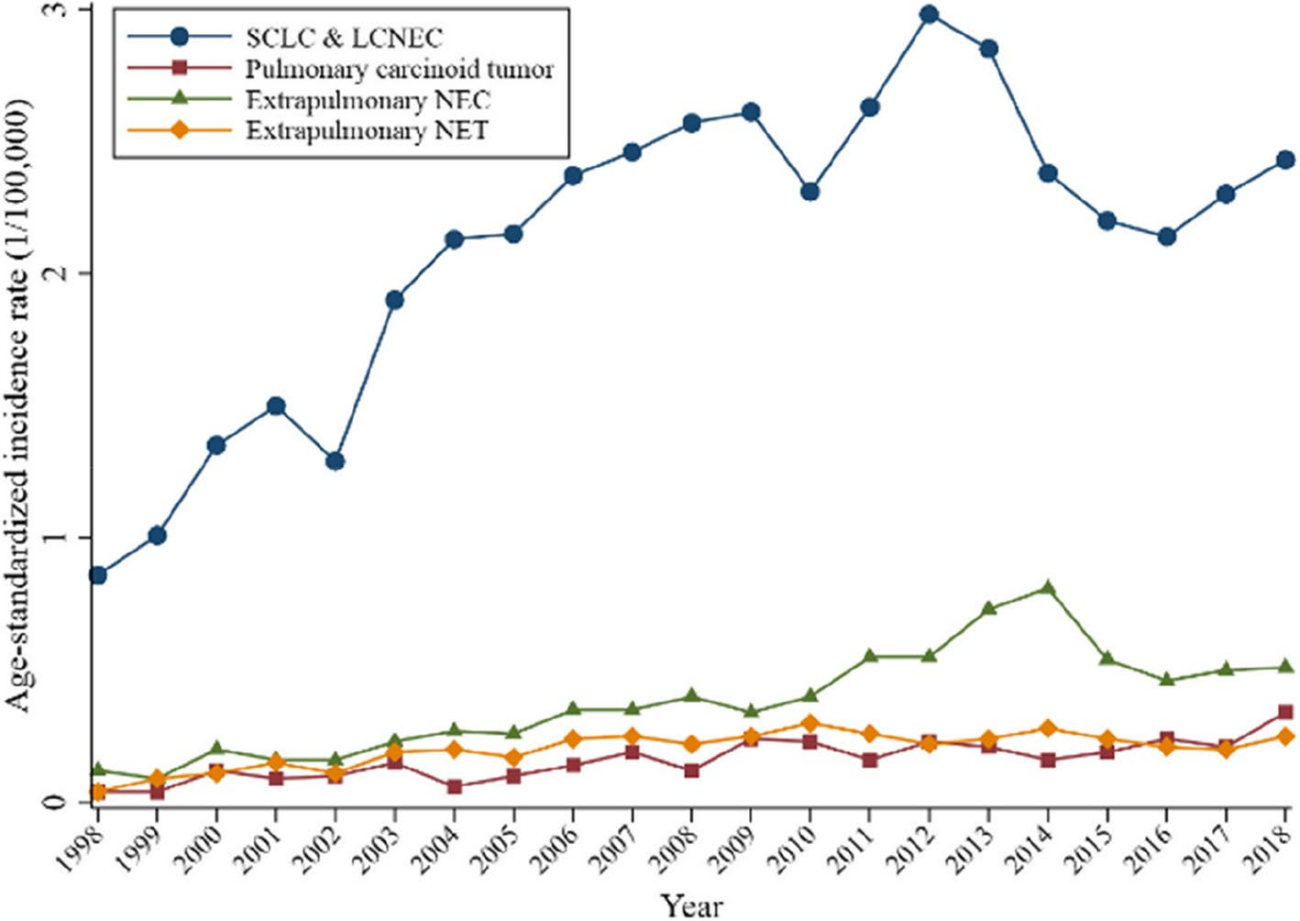
Incidence rate of NENs by histological type from 1998 to 2018 in Beijing, China. *Abbreviations* NEN, neuroendocrine neoplasm; NEC, neuroendocrine carcinoma; NET, neuroendocrine tumor; SCLC, small cell lung cancer; LCNEC, large cell neuroendocrine carcinoma

Statistics showed that the incidence of SCLC and LCNEC increased more rapidly from 1998 to 2008, then tended to decrease slightly from 2008 to 2018. The AAPC during these periods was 11.47% (95% CI: 8.75–14.25%) and − 1.44% (95% CI: − 3.50–0.66%). The overall trend in the incidence of pulmonary carcinoid tumor increased with the AAPC of ASIR at 11.41% (95% CI: 2.40–21.22%) in the first decade and 4.44% (95% CI: −0.94–10.11%) in the second decade. The trend of extrapulmonary NEC incidence was similar to that of SCLC and LCNEC, for which the AAPC was 14.22% (95% CI: 9.91–18.70%) and 3.26% (95% CI: − 2.04–8.85%), respectively. The incidence of extrapulmonary NET first showed a significant increase and then regional stability, with AAPC 15.45% (95% CI: 8.72–22.58%) and − 1.12% (95% CI: − 3.70–1.53%) (Fig. 3).

### Primary sites

According to the data (Table 3), the main primary sites of NENs included the lung (10,276 cases, accounting for 79.6%), stomach (400 cases, 3.1%), small intestine (102 cases, 0.8%), colon (156 cases, 1.2%), rectum (572 cases, 4.4%), pancreas (244 cases, 1.9%), and esophagus (228 cases, 1.8%). Other sites (918 cases, 7.1%) included the mediastinum, female reproductive system, and urinary system. The most common primary sites of NENs were lung (2.38/100,000) and rectum (0.14/100,000). Significantly more NENs originated from the lung than from other organs.

**Table 3:** Cases and age-standardized incidence rate of neuroendocrine neoplasms per 100,000 person-years in Beijing by primary tumor site, 1998–2018

Year	All	Lung	Stomach	Small Intestine	Colon	Rectum	Pancreas	Esophagus
1998	1.07(149)	0.90(128)	0.007 (1)	0.000(0)	0.023 (3)	0.034 (5)	0.013 (2)	0.014 (2)
1999	1.24(178)	1.06(152)	0.029 (4)	0.000(0)	0.005 (1)	0.054 (8)	0.005 (1)	0.027 (4)
2000	1.79(269)	1.47(221)	0.061 (9)	0.006 (1)	0.018 (2)	0.045 (7)	0.024 (3)	0.055 (8)
2001	1.90(295)	1.58(248)	0.006 (1)	0.014 (2)	0.027 (3)	0.056 (9)	0.064 (9)	0.06 (10)
2002	1.67(274)	1.39(230)	0.032 (5)	0.006 (1)	0.027 (3)	0.030 (5)	0.016 (2)	0.08 (12)
2003	2.46(409)	2.03(337)	0.034 (6)	0.022 (4)	0.042 (7)	0.07 (13)	0.054 (8)	0.056 (9)
2004	2.66(461)	2.18(381)	0.043 (8)	0.023 (4)	0.06 (12)	0.08 (11)	0.027 (5)	0.09 (14)
2005	2.68(490)	2.23(410)	0.043 (8)	0.020 (3)	0.010 (2)	0.10 (17)	0.042 (6)	0.07 (13)
2006	3.10(588)	2.49(474)	0.07 (15)	0.026 (4)	0.019 (4)	0.09 (16)	0.08 (15)	0.09 (18)
2007	3.25(636)	2.64(521)	0.08 (16)	0.008 (2)	0.042 (7)	0.12 (21)	0.027 (5)	0.05 (11)
2008	3.30(664)	2.68(544)	0.06 (13)	0.018 (4)	0.024 (4)	0.17 (32)	0.10 (18)	0.05 (11)
2009	3.44(730)	2.82(605)	0.09 (20)	0.013 (3)	0.019 (4)	0.16 (32)	0.042 (7)	0.06 (15)
2010	3.24(704)	2.52(555)	0.09 (23)	0.05 (10)	0.017 (4)	0.20(39)	0.08 (17)	0.04 (10)
2011	3.61(805)	2.78(630)	0.12 (27)	0.05 (10)	0.022 (5)	0.21(42)	0.11 (23)	0.06 (12)
2012	3.99(923)	3.20(746)	0.12 (28)	0.06 (13)	0.04 (10)	0.26(57)	0.09 (20)	0.04 (10)
2013	4.03(934)	3.05(717)	0.14(36)	0.05 (10)	0.06 (13)	0.30(62)	0.11 (23)	0.08 (18)
2014	3.63(884)	2.52(632)	0.19(48)	0.033 (8)	0.06 (15)	0.27(60)	0.11 (23)	0.05 (13)
2015	3.17(808)	2.38(622)	0.15(40)	0.023 (5)	0.06 (12)	0.18(38)	0.05 (11)	0.037 (9)
2016	3.06(821)	2.35(641)	0.10 (25)	0.024 (5)	0.05 (14)	0.14(35)	0.06 (14)	0.04 (10)
2017	3.21(881)	2.49(696)	0.11 (28)	0.033 (8)	0.06 (17)	0.14(33)	0.05 (13)	0.033 (9)
2018	3.53(993)	2.73(786)	0.14(39)	0.022 (5)	0.05 (14)	0.14 (30)	0.08 (19)	0.04 (10)
Total	3.01	2.38	0.09	0.03	0.04	0.14	0.06	0.05
1998-2018AAPC	5.08%	4.42%	12.82%	7.55%	7.82%	9.48%	9.15%	-0.21%
95% CI *P* value	3.35–6.84 < 0.001	2.69–6.18 < 0.001	8.29–17.55 < 0.001	2.27–13.10 0.007	3.48–12.34 0.001	6.24–12.81 < 0.001	4.19–14.35 0.001	-3.55-3.25 0.900

**Fig. 4:**
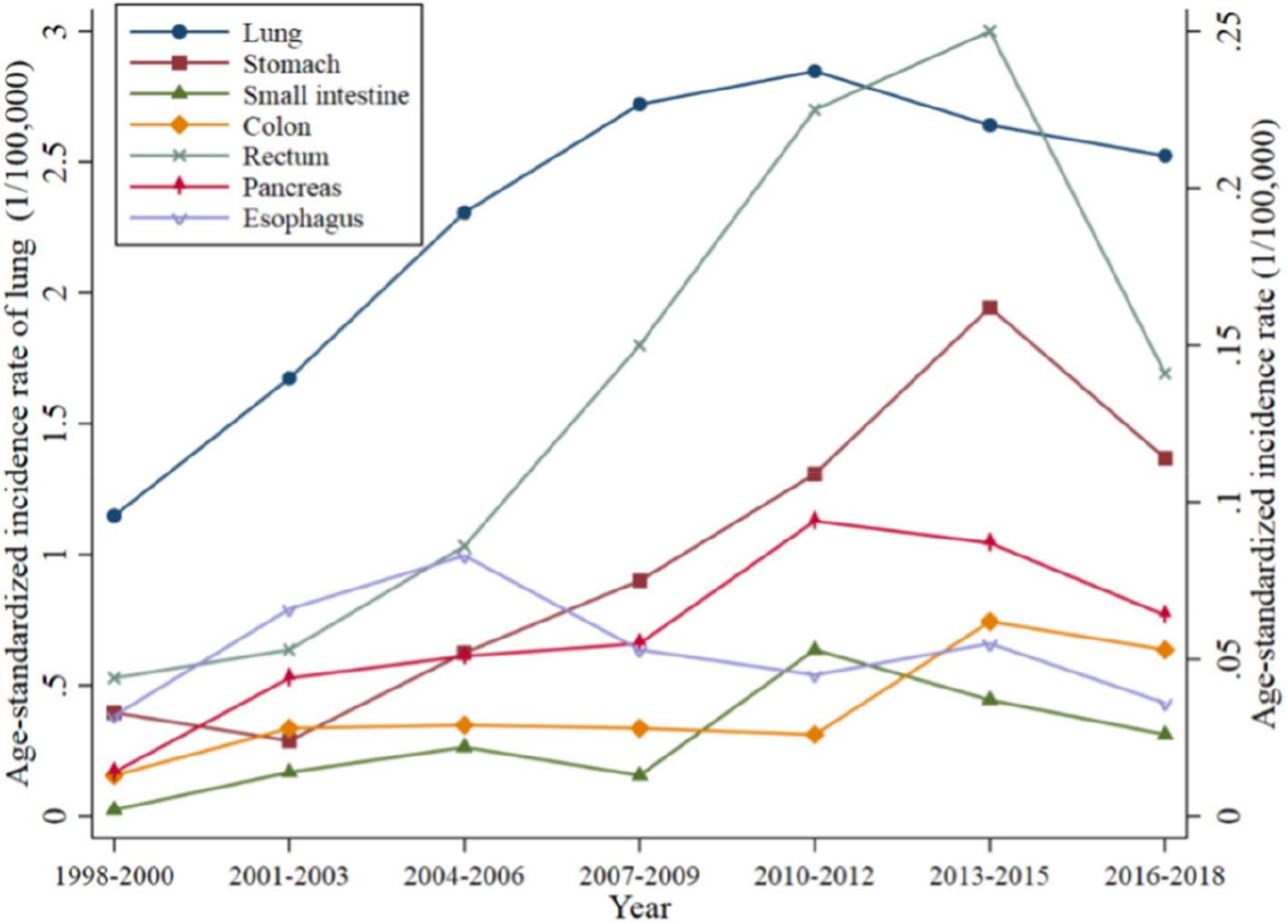
Age-standardized incidence rate of neuroendocrine neoplasms by primary site from 1998 to 2018 in Beijing, China

It can be observed that the incidence of NENs at different locations showed different characteristics. Although NENs from the lung, pancreas, and small intestine increased before 2010–2012, they showed a slow downward trend after that. The annual incidence of NENs from the rectum and stomach peaked during 2013–2015 and then showed a significant decreasing trend. The incidence of NENs from the colon peaked in 2013–2015 and remained stable. In contrast, esophageal NENs peaked earlier, in approximately 2004–2006, and then began to show a fluctuating trend of decreasing incidence (Fig. 4).

### Stage

Data for a total of 2538 patients with a definite stage were collected from 2012 to 2018, including 207 patients (8.01%) in stage I, 186 patients (7.20%) in stage II, 724 patients (28.03%) in stage III, and 1466 patients (56.76%) in stage IV. There were 393 patients (15.21%) in the early stage with combined staging (stage I and II) (Table 4). The proportion of patients diagnosed as having an advanced stage of SCLC and LCNEC was 89.04%; the proportion diagnosed as having an advanced stage of pulmonary carcinoid tumor was 65.15%; and the proportion of patients diagnosed as having an advanced stage of extrapulmonary NEC and NET was 73.85% and 53.97%, respectively. From 2012 to 2018, the proportion of all early-stage neuroendocrine tumors increased from 10.97% in 2012 to 22.22% in 2018 (χ2 = 18.92, *P* < 0.01). The proportion of patients with early-stage SCLC and LCNEC and extrapulmonary NEC increased from 8.21% to 18.75% in 2012 to 14.98% and 38.18% in 2018, respectively, and the trend was statistically significant.

**Table 4:** Stage distribution of patients with NENs by primary tumor site and year in Beijing, 2012–2018

	All NENs		SCLC&LCLC		Pulmonary carcinoid tumor		Extrapulmonary NEC		Extrapulmonary NET	
Year	Early	Advanced	Early	Advanced	Early	Advanced	Early	Advanced	Early	Advanced
2012	51(10.97)	414(89.03)	32(8.21)	358(91.79)	7(31.82)	15(68.18)	9(18.75)	39(81.25)	3(60.00)	2(40.00)
2013	63(13.94)	389(86.06)	37(10.11)	329(89.89)	8(38.10)	13(61.90)	15(25.00)	45(75.00)	3(60.00)	2(40.00)
2014	53(14.02)	325(85.98)	28(9.69)	261(90.31)	4(25.00)	12(75.00)	14(21.88)	50(78.13)	7(77.78)	2(22.22)
2015	43(14.63)	251(85.37)	23(10.70)	192(89.30)	8(38.10)	13(61.90)	11(22.00)	39(78.00)	1(12.50)	7(87.50)
2016	49(15.03)	277(84.97)	27(10.93)	220(89.07)	5(22.73)	17(77.27)	12(27.27)	32(72.73)	5(38.46)	8(61.54)
2017	60(17.91)	275(82.09)	37(14.07)	226(85.93)	4(36.36)	7(63.64)	15(30.00)	35(70.00)	4(36.36)	7(63.64)
2018	74(22.22)	259(77.78)	37(14.98)	210(85.02)	10(52.63)	9(47.37)	21(38.18)	34(61.82)	6(50.00)	6(50.00)
Total	393(15.21)	2190(84.79)	221(10.96)	1796(89.04)	46(34.85)	86(65.15)	97(26.15)	274(73.85)	29(46.03)	34(53.97)
Trend	Slope = 0.02 Chi for trend = 18.92 *p* < 0.01		Slope = 0.01 Chi for trend = 9.31 *p* < 0.01		Slope = 0.02 Chi for trend = 0.83 *p* = 0.36		Slope = 0.01 Chi for trend = 9.31 *p* = 0.02		Slope=-0.04 Chi for trend = 1.20 *p* = 0.27	

Values are no. cases (%), unless otherwise noted

*NEN* Neuroendocrine neoplasm, *SCLC* Small cell lung cancer, *LCNEC* Large cell neuroendocrine carcinoma, *NEC* Neuroendocrine carcinoma, *NET* Neuroendocrine tumor

### Survival

The median follow-up was 10.24 years. The 1-, 3-, 5-, and 10- year overall survival for patients with NENs was 54.42%, 30.10%, 26.31%, and 23.13%, respectively (Table 5). The OS differed significantly according to the histology subtype as well as by sex (*P* < 0.001; Table 5). The median OS time for all patients was 13.7 months. The median OS of female patients was 20.0 months (95% CI: 18.3–21.5 months), which was much longer than that of male patients (12.4 months; 95% CI: 12.1–12.8 months). There was no significant difference in the median OS time between urban and rural patients, with 13.8 months (95% CI: 13.3–14.4 months) versus 13.4 months (95% CI: 12.9–14.0 months).

**Table 5:** Survival analysis based on age, sex, residential area, and histology type in patients with NENs in Beijing, 1998–2018

Year	Total	Sex		Area		Cancer site			
		Male	Female	Urban	Rural	SCLC&LCNEC	Pulmonary carcinoid tumor	Extrapulmonary NEC	Extrapulmonary NET
1	54.42 (53.55–55.28)	51.51 (50.48–52.52)	61.69 (60.1-63.24)	54.39 (53.33–55.44)	54.48 (52.99–55.95)	48.13 (47.13–49.13)	68.34 (64.85–71.57)	65.82 (63.54-68)	91.23 (89.08–92.97)
2	36.13 (35.3-36.96)	31.93 (30.98–32.89)	46.61 (44.99–48.21)	37.32 (36.3-38.35)	33.79 (32.38–35.2)	27.51 (26.62–28.41)	55.16 (51.49–58.67)	51.86 (49.48–54.18)	86.47 (83.93–88.63)
3	30.10 (29.31–30.89)	26.10 (25.21–27.01)	40.07 (38.48–41.65)	31.40 (30.42–32.39)	27.55 (26.23–28.88)	21.04 (20.23–21.86)	48.75 (45.09–52.31)	46.59 (44.23–48.92)	84.38 (81.71–86.7)
4	27.69 (26.92–28.47)	23.65 (22.78–24.53)	37.77 (36.2-39.34)	29.01 (28.04–29.98)	25.10 (23.82–26.41)	18.70 (17.92–19.49)	46.22 (42.57–49.8)	43.44 (41.09–45.77)	82.85 (80.08–85.27)
5	26.31 (25.55–27.08)	22.40 (21.55–23.27)	36.05 (34.49–37.62)	27.58 (26.63–28.54)	23.81 (22.54–25.1)	17.55 (16.79–18.33)	44.37 (40.7-47.97)	41.34 (38.99–43.67)	80.78 (77.88–83.34)
6	25.48 (24.72–26.24)	21.77 (20.91–22.63)	34.73 (33.17–36.29)	26.57 (25.63–27.53)	23.33 (22.07–24.62)	16.97 (16.22–17.74)	43.14 (39.45–46.77)	39.46 (37.11–41.8)	79.41 (76.41–82.07)
7	24.71 (23.95–25.47)	21.04 (20.2–21.9)	33.84 (32.28–35.4)	25.69 (24.75–26.65)	22.79 (21.53–24.08)	16.41 (15.66–17.17)	41.10 (37.36–44.81)	38.16 (35.8-40.52)	78.43 (75.36–81.16)
8	24.15 (23.4-24.92)	20.51 (19.67–21.37)	33.23 (31.67–34.8)	25.07 (24.13–26.03)	22.37 (21.11–23.66)	16.08 (15.34–16.85)	39.55 (35.76–43.32)	36.90 (34.52–39.29)	77.32 (74.16–80.14)
9	23.54 (22.78–24.31)	20.02 (19.18–20.88)	32.31 (30.74–33.88)	24.37 (23.43–25.32)	21.95 (20.68–23.24)	15.65 (14.9–16.4)	38.4 (34.56–42.23)	35.83 (33.42–38.24)	76.08 (72.81–79.02)
10	23.13 (22.37–23.9)	19.54 (18.7–20.4)	32.09 (30.52–33.67)	23.87 (22.93–24.82)	21.75 (20.48–23.04)	15.36 (14.62–16.11)	38.06 (34.2-41.92)	34.93 (32.48–37.4)	75.17 (71.8-78.19)
		χ^2^ = 231.03, *P* < 0.01		χ^2^ = 6.96, *P* < 0.01		χ^2^ = 1365.79, *P* < 0.01			

Values in the table are % (range)

*NEN* Neuroendocrine neoplasm, *SCLC* Small cell lung cancer, *LCNEC* Large cell neuroendocrine carcinoma, *NEC* Neuroendocrine carcinoma, *NET* Neuroendocrine tumor

According to different pathological types, the median OS for patients with SCLC and LCNEC (11.5 months; 95% CI: 11.1–11.8 months) was significantly worse than that of patients with pulmonary carcinoid tumor or extrapulmonary NEC (33.5 months, 95% CI: 24.4–44.4 months; 26.8 months, 95% CI: 23.2–32.3 months, respectively). At the time of analysis, the median OS time of patients with extrapulmonary NET patients was not yet reached. The 5-year OS rate of pulmonary NENs was 19.45% (18.68–20.23%) and that of extrapulmonary NENs was 54.02% (52.05–55.95%). The 5-year survival was 21.19% (20.44–21.96%) for NEC and 63.60% (61.13–65.95%) for NET.

**Fig. 5:**
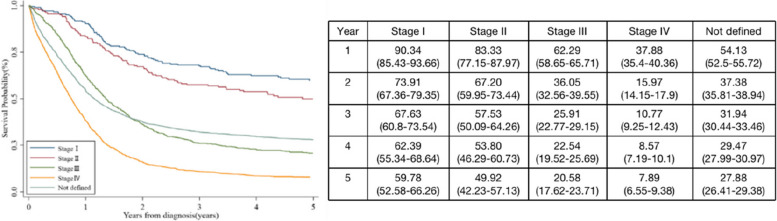
Survival curves and survival probability for different stages of neuroendocrine neoplasm

In terms of the overall population, the tumor stage at diagnosis was significantly correlated with OS. The more advanced the disease stage, the worse the survival. For example, the median OS time of stage I patients was not reached (95% CI, 90.59 months–not reached), with 5-year survival of 59.78% (95% CI, 52.58–66.26%); this was only 13.78 months (95% CI, 13.08–14.59 months), with 5-year survival of 27.88% (95% CI, 26.41–29.38%) for stage IV patients. This difference in survival of different stages was evident from the first year of diagnosis (Fig. 5).

**Fig. 6:**
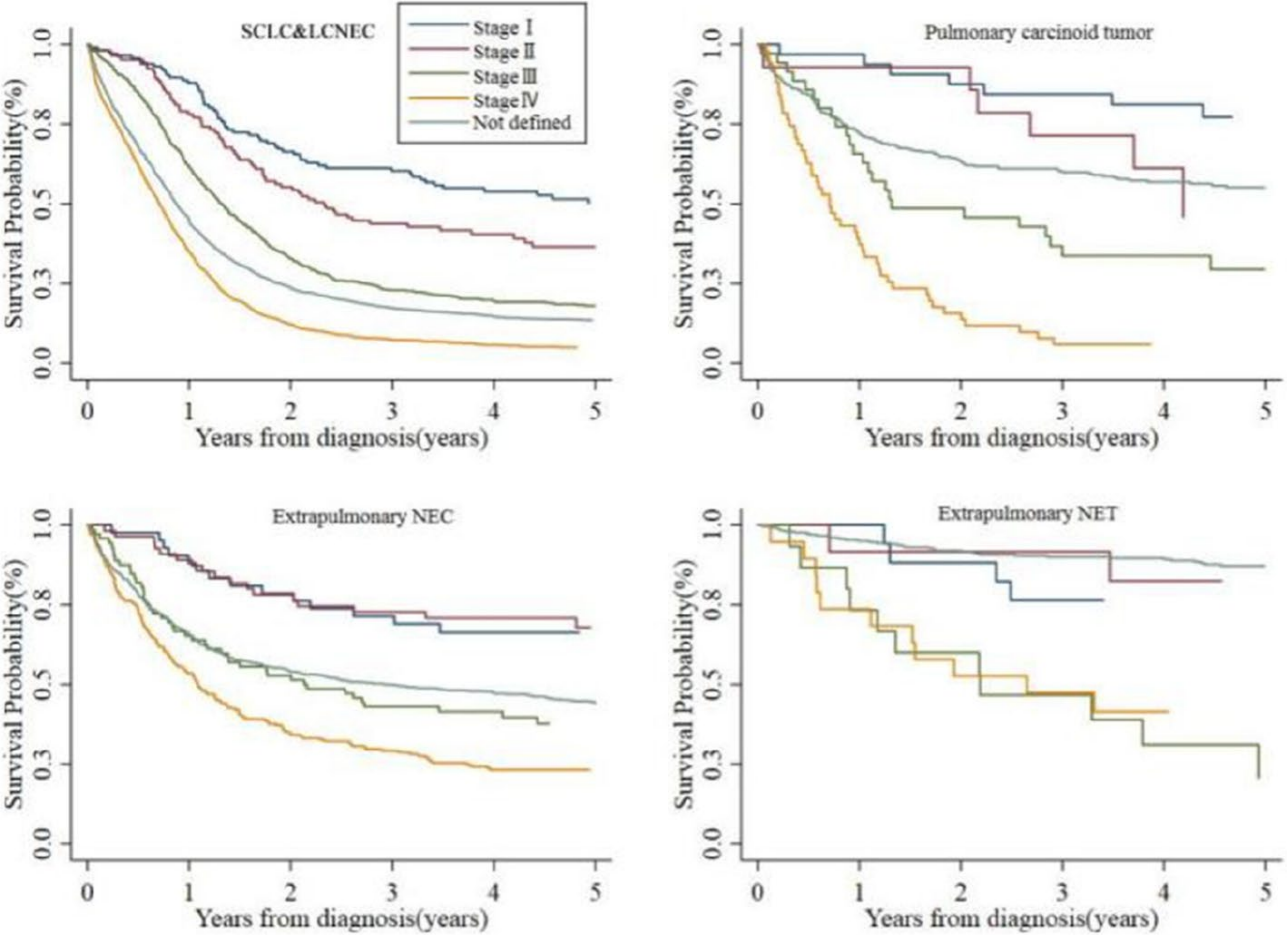
Survival curves for different histology types of neuroendocrine neoplasm in Beijing, China, 1998–2018

Clinical staging was significantly associated with OS in each pathological type (Fig. 6). From stage I to stage IV, the survival time of patients was gradually shortened. The 5-year survival rates were as follows: SCLC and LCNEC (stage I, 50.22%; stage II, 36.51%; stage III, 18.02%; stage IV, 4.89%), pulmonary carcinoid tumor (stage I, 77.27%; stage II, 45.92%; stage III, 29.46%; stage IV, 5.88%), extrapulmonary NEC (stage I, 66.29%; stage II, 67.86%; stage III, 37.68%; stage IV, 23.17%), and extrapulmonary NET (stage I, 76.47%; stage II, 82.50%; stage III, 20.74%; stage IV 41.45%).

**Fig. 7:**
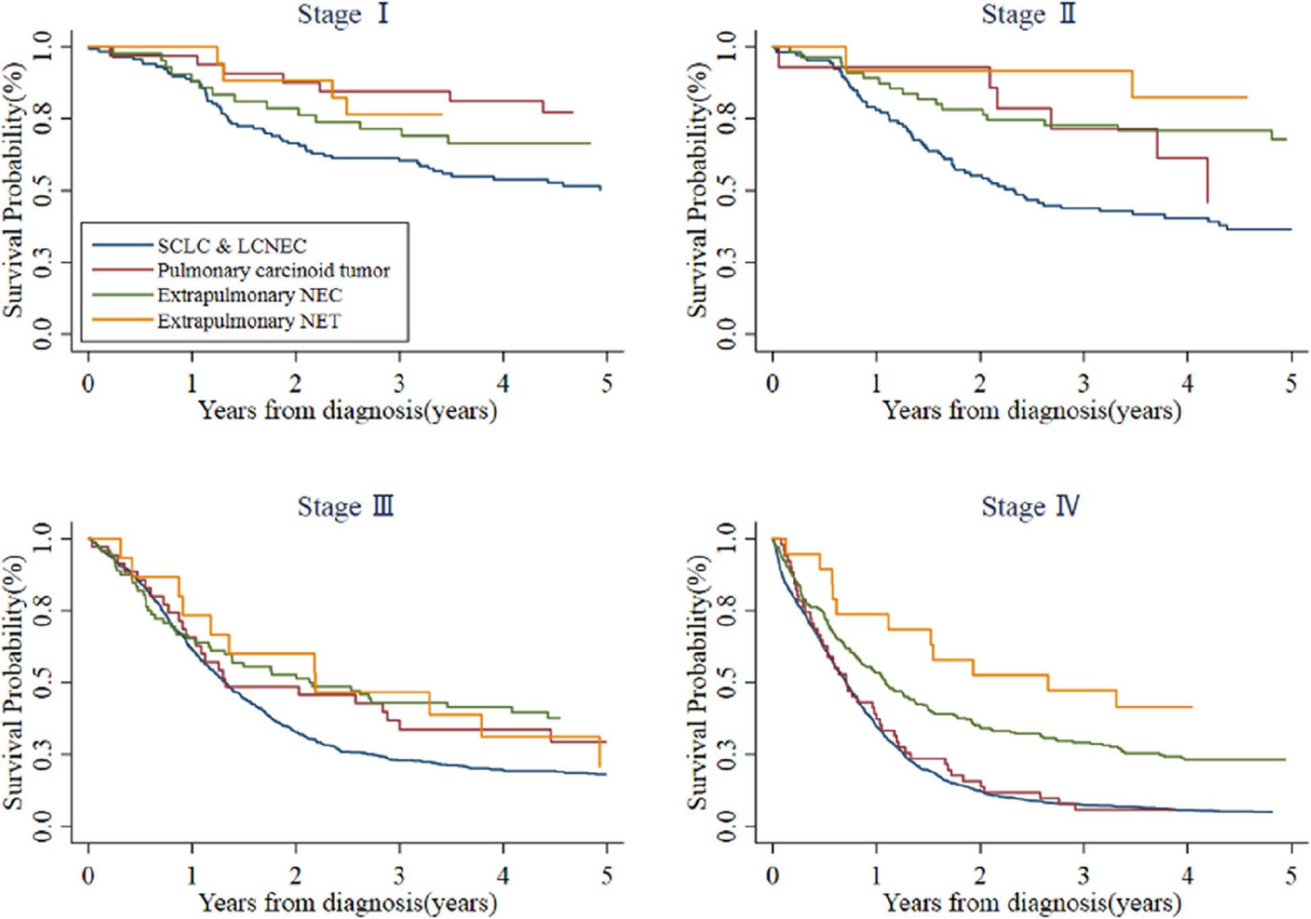
Survival curves for different stages of NEN in Beijing, China, 1998–2018. *Abbreviations* NEN, neuroendocrine neoplasm; NEC, neuroendocrine carcinoma; NET, neuroendocrine tumor; SCLC, small cell lung cancer; LCNEC, large cell neuroendocrine carcinoma

Within the same clinical stage, different history types had distinct survival rates (Fig. 7). There was a statistically significant difference in OS between SCLC and LCNEC and pulmonary carcinoid tumor at stage I (χ2 = 7.57, *P* < 0.01). At stage II, SCLC and LCNEC survival differed in comparison with extrapulmonary NEC (χ2 = 9.44, *P* < 0.01) and extrapulmonary NET (χ2 = 6.84, *P* < 0.01). For patients with stage III, only SCLC and LCNEC and extrapulmonary NEC (χ2 = 9.25, *P* < 0.01) differed in survival. In stage IV, the OS of SCLC and LCNEC was not significantly different from that of pulmonary carcinoid tumor, but both were significantly worse than survival of extrapulmonary NEC (SCLC and LCNEC vs. extrapulmonary NEC, χ2 = 55.87, *P* < 0.01; pulmonary carcinoid tumor vs. extrapulmonary NEC, χ2 = 11.28, *P* < 0.01) and extrapulmonary NET (SCLC and LCNEC vs. extrapulmonary NET, χ2 = 15.46, *P* < 0.01; pulmonary carcinoid tumor vs. extrapulmonary NET, χ2 = 11.56, *P* < 0.01). Generally, SCLC and LCNEC had worst prognosis whereas extrapulmonary NET had favorable survival.

## Discussion

In this population-based study, we found that from 1998 to 2018, the incidence rate of NENs in Beijing showed a significant continuous increasing trend, which was the same as reports from other countries [1, 8, 12] This increase was partly owing to improvements in the classification of these tumors and the increasing awareness of NENs by physicians and pathologists. Also, widespread use of endoscopy, sonography, computed tomography (CT) scan, and needle biopsy in cancer screening contributed to an increase in the reported incidence of NENs [1]. Advancement in somatostatin receptor imaging and theragnostic approaches using peptide receptor radionuclide therapy (PRRT) have also changed the paradigm of diagnosis and management of neuroendocrine tumor in recent years, although these new techniques were not widely used during a certain period (1998–2018) of this study. Additionally, cancer registration in China has made great progress [13], which has been helpful in collecting more useful data. However, when standardized by age of the world standard population, the incidence of NENs in Beijing showed a significant increasing trend in the first 15 years, up to 4.03 per 100,000 in 2013; this then began to plateau after 2013. The possible reasons for this phenomenon may include aging of the population in Beijing [14] and the WHO pathological classification being updated in 2010 [15].

We found that the ASIRW was significantly higher in male patients than in female patients, similar to prior results showing that male patients comprised a larger proportion of NENs. Yao et al. reported that the incidence of NETs was 5.35 per 100,000 population among male individuals versus 4.76 per 100,000 among female patients in 2000–2004, according to the Surveillance, Epidemiology, and End Results (SEER) database [1]. In Japan, Masui et al. reported that the overall incidence of NENs in 2016 among male individuals was 2.09 per 100,000 population and that in female patients was 1.39 per 100,000 population [9]. Studies by Hauso et al. showed the total incidence of NETs was higher in men (male to female ratio, 1.1–1.2) in Norway [16]. Fan et al. found that the sex ratios for different anatomic sites varied greatly, with male patients mostly having cardiac and gastric body NEN and a smaller number having rectal NEN; female patients predominantly had pancreatic NENs [12]. In our study population, this may be owing to the group with SCLC and LCNEC accounting for three-quarters of patients; male patients also accounted for three-quarters in this group. The main risk factor for lung cancer is smoking [1]; in China, most smokers are men and women rarely smoke. This could possibly explain why more male patients were diagnosed in the total population whereas the incidence rate of extrapulmonary NECs and NETs was unrelated to sex.

Cheung et al. noted that patients living in rural areas experienced a higher incidence of NETs [17]. In a Canadian retrospective cohort study, rural residence and low socioeconomic status were found to be independent predictors of survival in adult patients with NETs [18]. Gosain also reported that incidence rates of NETs were higher in rural than urban areas [19]. In our study, we found that the incidence of NENs in rural areas was significantly lower than that in urban areas at first; this then increased at a very high rate, which exceeded and remained higher than the rate in urban areas during 2008–2018. This may be related to the rapid development of the economy and transportation in the Beijing area over the past 21 years. The diagnosis of diseases such as NENs is complex and requires technical and medical teams in tertiary hospitals. However, tertiary hospitals in Beijing are concentrated in urban areas, and it was previously relatively difficult for patients in rural parts of the country to visit a doctor. That may lead to the illusion that the incidence of NENs in rural areas was lower than that in urban areas in the past. However, real factors affecting the difference between urban and rural areas remain to be elucidated in further studies.

A Japanese study [9] suggests that the age distributions differ within GEP-NENs, according to the primary site. We found that the median age of patients with SCLC and LCNEC, pulmonary carcinoid tumor, and extrapulmonary NEC was approximately 63–65 years, which was similar to data reported in other countries [20]. However, when compared with other NENs, extrapulmonary NETs appear at a slightly younger age. This suggests that the causes of these diseases may be different and need to be further explored.

Different from previous studies [20–22], our data included patients with SCLC and LCNEC, which resulted in the most common primary tumor site in our study being the lung, accounting for nearly 80% of the total population, and the main pathological type was NEC, not NET. There are many possible reasons for the observed changes in incidence. First, more tumors may be found incidentally over time. Rates of non-invasive examinations like CT scan in the general population have been steadily increasing, which is an important means of lung tumor discovery [23]. Second, screening for lung cancer appears to be rising [24]. As screening rates increase, more lung NENs may be detected. However, finding tumors of gastrointestinal origin often requires the presence of symptoms and an invasive procedure, such as gastroscopy or colonoscopy, which are often refused by patients out of fear. Additionally, high exposure levels to carcinogenic factors like environmental pollution, smoking, and tuberculosis infection may be important factors in the high incidence of respiratory tract tumors in China. These reasons may partially explain why the incidence of NENs of the lung is much higher than that of other sites.

Interestingly, most reports mention that fewer NETs originate in the stomach than the small intestine whereas our results show more NENs originating in the stomach than the small intestine. Coincidentally, this feature was also found in Fan’s study [12]. Yao reported that among Asians/Pacific Islanders in North America, the incidence of rectal NENs was approximately five times that of small intestinal, pancreatic, and gastric NENs, but these rates were not observed in Whites or Blacks [1]. These differences were owing in part to racial disparities. However, small intestinal NENs are always asymptomatic. The small intestine is an organ that is difficult to access with inferior radiographic and endoscopic techniques. These reasons make the diagnosis of small intestine tumors more difficult than tumors of other sites, resulting in a detection rate that may be lower than the actual incidence. Additionally, previous studies have shown that the incidence of upper digestive tract NENs may be related to economic level, nutrition state, and dietary habits [12, 19, 25]. These factors could partially explain the higher incidence of NENs in the stomach found in our study.

In recent years, with deepening understanding of cancer risk factors, the rise in early screening for high-risk groups [24], and the change to healthier lifestyle habits, the incidence of NENs in the lung and digestive tract has been stable or decreased to varying degrees. This is a welcome change, which indicates that some pathogenic factors related to these tumors can be avoided [26]; this also shows that public health education on cancer prevention and treatment has had a certain positive role.

The stage of NENs at diagnosis was found to be closely related to survival. A later stage predicts worse prognosis. A study in the United States (US) showed that the median survival time of patients with NENs was 9.3 years; the median survival time of these patients with an earlier stage was longer, often more than 30 years. The median survival time of patients with NENs who have regional metastasis and distant metastasis was significantly shorter, at 10 years and 12 months, respectively [21]. In Canada, the 10-year survival rates of patients with earlier-stage NENs and distant metastasis were 68.2% and 17.5%, respectively [18]. Accurate staging of NENs is necessary for clinicians to evaluate the disease, formulate a reasonable diagnosis and treatment strategy, and predict the prognosis of patients. In our study, most patients were already at an advanced stage when diagnosed, accounting for approximately 85% of our study population. The proportion of patients with advanced NENs was much higher than the 20.1% in the aforementioned US study [21], with a median survival time of all patients with NENs in our study of only 13.6 months. For our patients with NENs and distant metastasis, the median survival time was only 9 months, which was also shorter than the 12 months in the US study [21]. These large differences highlight that there is still a need to raise awareness about these diseases and further promotion of cancer screening is essential. Improvement in the rate of diagnosis at an early stage will help to improve the survival rate and prolong survival time. Concerning the survival data of patients with the same stage, a gap remains between China and developed countries in terms of continuous improvement in treatment outcomes. The development of drugs such as mammalian target of rapamycin (mTOR) inhibitors and anti-angiogenic tyrosine kinase inhibitors (TKIs) may bring about greater patient benefit. PRRT has emerged as a crucial strategy for treating advanced, unresectable neuroendocrine tumors, considerably changing the prognosis of these diseases.

We found that male patients had worse survival than female patients. A study using the SEER database divided 20,836 patients with NENs into groups according to stage, degree of differentiation, age, and other factors and found that the prognosis of female patients was significantly better than that of male patients in each subgroup [27]. In Taiwan, China, the 5-year and 10-year survival rates of female patients with NENs were 62.6% and 53.5% whereas those of male patients were only 49.0% and 39.7%, respectively [8]. Several studies have shown that the expression of estrogen and progesterone receptors is related to the prognosis of patients with NENs, and those with negative receptor expression tend to have a poor prognosis; this finding provides a biological basis for the difference in prognosis by sex [28, 29]. Because most patients with NENs have a high degree of tumor differentiation, showing slow growth and a relatively long survival time, some patients die owing to diseases other than NENs, including secondary primary tumors and non-cancer related diseases. Compared with female patients, male patients have a greater burden of comorbidities and a higher probability of non-cancer death [30]. Thus, these confounders should not be ignored when looking at the difference in prognosis according to sex.

We found that NENs originating from the lung had worse OS than extrapulmonary NENs (5-year OS: 19.45% vs. 54.02%), and NECs had worse OS than NETs (5-year OS: 21.19% vs. 63.60%). Many studies have suggested that the survival of patients with NENs varies greatly according to different primary sites and tissue types. In the US, the prognosis of NENs from the rectum and appendix was the best, with a median survival time of 24.6 years and 30.0 years, respectively. The prognosis of pancreatic and pulmonary NENs was poor, with a median survival time of 3.6 years and 5.5 years, respectively [21]. The prognosis of NENs in the rectum and appendix was also found to be the best in the study in Taiwan, with 5-year survival rates of 86.0% and 76.2%, respectively, whereas the 5-year survival rate for pulmonary NENs was only 32.6% [8]. According to the origin of embryonic development, pathogenic sites are divided into the foregut, midgut, and hindgut. We found that NENs from the hindgut had the best prognosis (*n* = 990, OS: not reached; 10-year OS: 66.79%). NENs from the foregut had the worst prognosis (*n* = 11,460, OS: 12.3 months, 95% CI: 12–12.6; 10-year OS: 18.77%), and NENs from the midgut had an intermediate prognosis (*n* = 124, OS: 102.1 months, 95% CI: 29.4–not reached; 10-year OS: 49.96%) (Supplement 1). Such results coincide with those reported in prior studies, implying the important influence of the primary site on survival.

For patients with NENs in the same site, the prognosis with different histology types is also significantly different. For example, in lung NENs, the median survival time of patients with SCLC and LCNEC is 9 months and 10 months whereas that of patients with typical carcinoid and atypical carcinoid is 209 months and 104 months, respectively [31]. In our study, the median survival time of patients with SCLC and LCNEC was 11.5 months whereas that of pulmonary carcinoid tumor was 33.5 months. In the future, a nomogram model integrating sex, age group, site, histology type, and treatment information will contribute the prediction of NEN survival. Further improvement in the understanding and diagnosis of this large group of diseases is needed, and the therapeutic exploration in this field needs to be further deepened.

Recently, Zheng et al. reported the incidence and survival statistics of NENs in China as compared with those in the United States [32]. Compared with patients in the United States, Zheng’s study suggested that the incidence of NENs in China was lower, the growth rate was faster, and the prognosis was worse. Although those authors included patients from more provinces than Beijing, our data were more comprehensive in terms of pathological types. By including SCLC and LCNEC in the analysis, the results can more accurately reflect the heterogeneity, pathogenesis, and different prognostic characteristics of NENs.

### Limitations

First, all data in our study were re-abstracted to improve coding accuracy and data quality. However, the original pathological samples were not reviewed owing to a lack of resources. Second, the start and development of the medical system in China has occurred later than those in foreign countries. So far, a unified large-scale standardized medical database like the SEER database has not been successfully established. Therefore, we lacked detailed data such as environmental factors, income levels, lifestyle habits, histologic grade, concomitant diseases, and treatment plans, among others. It was therefore difficult to conduct an in-depth analysis of the influencing factors that may have led to the current results. Third, specific information on treatment in our retrospective data was limited to accurate analyses of survival prognostic factors; the survival prognostic information cannot be interpreted based on the changes in clinical treatment. However, at present, we are gradually improving this database system. In future research, it will be easier to explore topics worth further study and address more clinical problems.

## Conclusion

This was the first report of the epidemiological characteristics of NENs based on a retrospective population-based analysis. As the largest and most authoritative population-based epidemiological information regarding NENs in Beijing, China to date, this study will contribute to the diagnosis and management of these diseases. We anticipate further studies to explain the clinical and survival characteristics of NENs and improve patients’ prognosis.

### Supplementary Information

Supplementary Material 1.

## Data Availability

The dataset generated and analyzed during the current study is not publicly available owing to data protection regulations in the study country aiming to protect the privacy of study participants. Deidentified data are available from the corresponding author on reasonable request

## Abbreviations

NEN: Neuroendocrine Neoplasm
NET: Neuroendocrine Tumor
NET: Neuroendocrine Carcinomas
GEP-NEN: Gastroenteropancreatic Nens
IARC/IACR: International Agency for Research on Cancer/International Association of Cancer Registries
BCR: Beijing Cancer Registry
ICD-O-3.2: International Classification of Diseases for Oncology 3rd Edition
Beijing CDC: Beijing Center for Disease Prevention and Control
SCLC: Small Cell Lung Carcinoma
LCNEC: Pulmonary Large Cell Neuroendocrine Carcinoma
AJCC: American Joint Committee on Cancer
OS: Observed Survival
AAPC: Average Annual Percentage Change
ASIRW: Age-Standardized Incidence Rate by World standard population
CI: Confidence Interval
CT: Computed Tomography
WHO: World Health Organization
SEER: Surveillance, Epidemiology, and End Results
US: United States
PRRT: Peptide Receptor Radionuclide Therapy
