# Supplementary material for: Epidemiological characteristics of neuroendocrine neoplasms in Beijing: a population-based retrospective study
Source: BMC Public Health. 2024 May 24;24:1396. doi: 10.1186/s12889-024-18845-8 (PMC11127419; doi:10.1186/s12889-024-18845-8)
Supplement: Supplementary file 1 — Supplementary Material 1. [file 12889_2024_18845_MOESM1_ESM.docx]

**Supplementary 1** Survival Analysis Based on embryonic digestive tube of NENs in Beijing, China 1998-2018

| Year | Foregut | Midgut | Hindgut |
| --- | --- | --- | --- |
| 1 | 51.17(50.25-52.08) | 73.39(64.67-80.28) | 85.86(83.53-87.88) |
| 2 | 31.87(31.02-32.73) | 59.68(50.5-67.7) | 78.28(75.57-80.72) |
| 3 | 25.51(24.71-26.31) | 58.87(49.69-66.94) | 74.92(72.10-77.51) |
| 4 | 23.05(22.28-23.83) | 58.04(48.85-66.16) | 73.43(70.55-76.07) |
| 5 | 21.75(20.99-22.51) | 56.31(47.1-64.53) | 71.12(68.16-73.86) |
| 6 | 20.96(20.21-21.72) | 53.55(44.31-61.94) | 70.11(67.10-72.90) |
| 7 | 20.21(19.46-20.97) | 53.55(44.31-61.94) | 69.13(66.08-71.97) |
| 8 | 19.70(18.96-20.46) | 51.28(41.91-59.86) | 68.68(65.60-71.54) |
| 9 | 19.12(18.37-19.87) | 49.96(40.50-58.70) | 67.92(64.78-70.84) |
| 10 | 18.77(18.03-19.53) | 49.96(40.50-58.70) | 66.79(63.55-69.82) |
